# Macrophage susceptibility to infection by Ghanaian *Mycobacterium tuberculosis* complex lineages 4 and 5 varies with self-reported ethnicity

**DOI:** 10.3389/fcimb.2023.1163993

**Published:** 2023-08-14

**Authors:** Stephen Osei-Wusu, John K. A. Tetteh, Abdul Basit Musah, Desmond Opoku Ntiamoah, Nelly Arthur, Abraham Adjei, Ainhoa Arbues, Ebenezer Addo Ofori, Kwadwo Akyea Mensah, Sutaya Elsie Afua Galevo, Abena Frema Frempong, Prince Asare, Adwoa Asante-Poku, Isaac Darko Otchere, Kwadwo Asamoah Kusi, Tobias L. Lenz, Sebastien Gagneux, Damien Portevin, Dorothy Yeboah-Manu

**Affiliations:** ^1^ Noguchi Memorial Institute for Medical Research, University of Ghana, Legon, Ghana; ^2^ West African Centre for Cell Biology of Infectious Pathogens (WACCBIP), University of Ghana, Legon, Ghana; ^3^ Department of Chest Diseases, Korle-Bu Teaching Hospital, Accra, Ghana; ^4^ Swiss Tropical and Public Health Institute, Allschwil, Switzerland; ^5^ University of Basel, Basel, Switzerland; ^6^ Research Group for Evolutionary Immunogenomics, Department of Biology, University of Hamburg, Hamburg, Germany

**Keywords:** macrophage infection, Ghana, West Africa, tuberculosis, Ewe, Akan, ethnicity, *Mycobacterium tuberculosis* complex

## Abstract

**Background:**

The epidemiology of *Mycobacterium tuberculosis* complex (MTBC) lineage 5 (L5) infections in Ghana revealed a significantly increased prevalence in Ewes compared to other self-reported ethnic groups. In that context, we sought to investigate the early phase of tuberculosis (TB) infection using *ex vivo* infection of macrophages derived from the blood of Ewe and Akan ethnic group volunteers with MTBC L4 and L5 strains.

**Methods:**

The study participants consisted of 16 controls, among which self-reported Akan and Ewe ethnicity was equally represented, as well as 20 cured TB cases consisting of 11 Akans and 9 Ewes. Peripheral blood mononuclear cells were isolated from both healthy controls and cured TB cases. CD14^+^ monocytes were isolated and differentiated into monocyte-derived macrophages (MDMs) before infection with L4 or L5 endemic strains. The bacterial load was assessed after 2 hours (uptake) as well as 3 and 7 days post-infection.

**Results:**

We observed a higher capacity of MDMs from Ewes to phagocytose L4 strains (p < 0.001), translating into a higher bacillary load on day 7 (p < 0.001) compared to L5, despite the higher replication rate of L5 in Ewe MDMs (fold change: 1.4 *vs.* 1.2, p = 0.03) among the controls. On the contrary, within macrophages from Akans, we observed a significantly higher phagocytic uptake of L5 (p < 0.001) compared to L4, also translating into a higher load on day 7 (p = 0.04). However, the replication rate of L4 in Akan MDMs was higher than that of L5 (fold change: L4 = 1.2, L4 = 1.1, p = 0.04). Although there was no significant difference in the uptake of L4 and L5 among cured TB cases, there was a higher bacterial load of both L4 (p = 0.02) and L5 (p = 0.02) on day 7 in Ewe MDMs.

**Conclusion:**

Our results suggest that host ethnicity (driven by host genetic diversity), MTBC genetic diversity, and individual TB infection history are all acting together to modulate the outcome of macrophage infections by MTBC.

## 1 Introduction

Tuberculosis (TB) remains a global health challenge irrespective of the control measures and the advances made since the World Health Organization (WHO) declared TB a global health emergency in 1993 (WHO, 2021). TB has been the leading cause of death from a single infectious agent worldwide until the recent mortality associated with the SARS-CoV-2 pandemic in 2019 (WHO, 1994; WHO, 2021). It is estimated that approximately 10.6 million people fell ill with TB and 1.6 million died from the disease in 2021. The TB epidemic in Ghana reported an incidence of 136 per 100,000 in 2021 (WHO, 2022).

TB is caused by *Mycobacterium tuberculosis* complex (MTBC), which comprises 11 closely related species, some of which transmit preferentially within specific host genetic groups, while others have spread globally (Gagneux and Small, 2007; Stucki et al., 2016). Phylogenetic analysis of the major human-adapted MTBC (hMTBC) species classifies them into seven lineages (L1–7). Nonetheless, two additional lineages, L8 and L9, were recently discovered and mostly sporadically isolated in East Africa (NGabonziza et al., 2020; Coscolla et al., 2021). Phylogeographic distribution analysis revealed that lineage 4 in particular is globally distributed whereas other hMTBC lineages are mostly restricted to specific geographic areas (Comas et al., 2013; Stucki et al., 2016). TB epidemiology in Ghana is relatively unique for hosting six out of the nine known hMTBC lineages (de Jong et al., 2010b; Asante-Poku et al., 2016; Yeboah-Manu et al., 2016). Nevertheless, hMTBC lineages 5 and 6 remained restricted to West Africa for reasons not well understood. L5 and L6 MTBC strains constitute important human pathogens in West Africa, causing approximately 40% of TB cases in the region and approximately 20% of all TB cases in Ghana (de Jong et al., 2010b; Yeboah-Manu et al., 2016). Studies have reported a specific association of L5 infections with the Ewe ethnic group and L6 with HIV patients (de Jong et al., 2005; Asante-Poku et al., 2015; Asante-Poku et al., 2016). While a lower virulence of L6 isolates could support the reported association with HIV infection, the underlying reasons behind the association of L5 infection with specific ethnic groups remain puzzling and unresolved (de Jong et al., 2010a; Brites and Gagneux, 2015). A better understanding of this phenomenon would enlighten us on the underlying reasons driving the specific phylogeographic distribution of MTBC lineages in West Africa and may also support improved control strategies of L5 infections in particular.

MTBC is a successful pathogen due to its ability to invade and replicate within host cells in the lungs. It is transmitted following inhalation of infectious droplets from an infected person by an uninfected person (Sia and Rengarajan, 2019). Transmission may therefore occur through coughing, sneezing, or even talking and singing (Sia and Rengarajan, 2019). Upon inhalation, MTBC bacilli might interact with throat epithelial cells that aid their migration through the trachea to the alveoli (Fernández Tena and Casan Clarà, 2012; Torrelles and Schlesinger, 2017). Within the lung alveoli, bacilli are phagocytosed by alveolar macrophages that migrate to the parenchyma and generate an inflammatory response, leading to the chemotactic attraction of other immune cells and the generation of granulomas, multicellular structures encapsulating the infectious bacilli (Scriba et al., 2017).

The presence of MTBC in the alveoli leads to different outcomes: 1) bacilli may be directly eliminated by innate immune responses before the onset of adaptive immunity and memory of exposure, 2) MTBC infections are controlled and walled off within granulomatous responses potentially leading to latent infections, or 3) immunity is unable to control MTBC replication, and active TB disease ensues (Scriba et al., 2017; Sia and Rengarajan, 2019). These different outcomes depend on intricate host–pathogen interactions. While MTBC’s genetic background may influence its pathogenicity, the host-specific immune response also plays an important role in the outcome of the disease (Gagneux et al., 2006; Coscolla and Gagneux, 2010; Gagneux, 2012). Macrophages are at the forefront of the battle against MTBC bacilli invading the lung (Scriba et al., 2017). Macrophages are not only phagocytic cells, but they can also regulate important cellular processes such as tissue homeostasis, repair, and wound healing (Wynn and Vannella, 2016; Sreejit et al., 2020). Hence, the role of macrophages is central to the pathogenesis of a variety of infectious and non-infectious inflammatory diseases.

Previous research has shown the impact of ethnicity, gender, and nutrition on innate immune cell function including macrophages (Nahid et al., 2018). In conjunction with MTBC genetic factors associated with inflammatory responses of macrophages (Portevin et al., 2011), heterogeneity in macrophage functions that are genetically encoded may have been evolutionarily selected to influence TB disease outcome (Fan et al., 2023). We hypothesized that the association of L5 infections with the Ewe ethnic group and L4 with the Akan ethnic group may be supported by inherent variation in macrophage responses to different MTBC lineages. Therefore, the study was designed to characterize the early phase of L4 and L5 infections of macrophages derived from the blood of volunteers belonging to the self-reported Ewe or Akan ethnic group.

## 2 Materials and methods

### 2.1 Ethical consideration

Ethical clearance was obtained from the Institutional Review Boards (IRB) of Korle-Bu Teaching Hospital (KBTH) and Noguchi Memorial Institute for Medical Research (NMIMR), University of Ghana (Federal wide assurance number: FWA00001824). The study protocol was explained to all participants, and written informed consent was obtained from participants voluntarily. A questionnaire was then administered to the participants to obtain their demographic data.

### 2.2 Study design

This case–control study resulted from the prospective collection of blood samples between January 2020 and September 2020 and a retrospective inclusion of MTBC isolates characterized previously (Yeboah-Manu et al., 2016; Asare et al., 2018). The study was conducted at the Department of Chest Diseases of the Korle-Bu Teaching Hospital, and the Departments of Bacteriology and Immunology of NMIMR, University of Ghana.

### 2.3 Inclusion and exclusion criteria

Participants belonging to either the Akan or Ewe ethnic group who had successfully completed treatment of TB caused by MTBC L4 or L5 and confirmed to be without an active TB infection were recruited as cured TB case participants. The control group was composed of healthy individuals without a history of TB and above the age of 15 years. Since host genetic analysis was not carried out to confirm the ethnicity of the participants, only individuals with both parents belonging to a specific ethnic group were included in the study. Only individuals with hemoglobin levels above 10 g/dL, systolic blood pressure between 100 and 130 mm Hg, and diastolic pressure between 70 and 90 mm Hg were recruited. Individuals with TB co-morbidities (such as HIV, diabetes, and cancer), pregnant women, and persons on anti-depressant drugs were excluded from the study.

### 2.4 Blood sample collection

Before phlebotomy, blood pressure was monitored using an OMRON monitor (OMRON Healthcare Co., Ltd., Kyoto, Japan), hemoglobin levels were assessed using HemoCue Hb 201+ (HEMOCUE AB, Ängelholm, Sweden), and random blood sugar levels were measured using OneTouch Select Plus Simple glucometer (LifeScan, Malvern, PA, USA). For women, a pregnancy test was carried out using the One-Step Pregnancy Test (Hubei Meibao Biotechnology Co., Ltd., Hubei, China). Venous blood samples ranging between 30 and 50 mL were collected with 10-mL Vacutainer blood collection tubes containing sodium heparin (BD Biosciences, San Jose, CA, USA) and inverted multiple times to prevent blood clotting. The tubes were transported at room temperature within 4 hours of blood sample collection to the laboratory for peripheral blood mononuclear cell (PBMC) isolation.

### 2.5 Isolation of peripheral blood mononuclear cells

Blood diluted twofold in Roswell Park Memorial Institute (RPMI) 1640 (with l-glutamine and penicillin–streptomycin) was gently overlayed on Ficoll-Paque™ under sterile conditions and centrifuged at room temperature for 10 minutes at 400×*g* without breaks. The top plasma layer was carefully pipetted away, and mononuclear cells were collected and washed twice with RPMI–5% fetal bovine serum (FBS). PBMCs were counted using the Countess II automated cell counter and cryopreserved in liquid nitrogen at 20 million cells per mL in a cryo-vial until further analyses.

### 2.6 CD14 monocyte isolation, macrophage differentiation, and harvest

Cryopreserved PBMCs were briefly thawed in a 37°C water bath and washed twice with 10 mL of pre-warmed RPMI–10% FBS and benzonase at 0.0125 U/µL final. PBMCs were rested for 2 hours at 37°C/5% CO_2_. Monocyte isolation was performed using CD14 magnetic beads following the manufacturer’s recommendations (Miltenyi Biotec Swiss AG, Solothurn, Switzerland). CD14 monocytes were counted and differentiated into macrophages for 6 days at 37°C and 5% CO_2_ into tissue culture-treated dishes (Verreck et al., 2004) in the presence of human granulocyte–macrophage colony-stimulating factor (Thermo Fisher Scientific Inc., Waltham, MA, USA) at a final concentration of 10 ng/mL and a cell density of 8 × 10^5^ monocytes/cm^2^. The monocyte-derived macrophage (MDM) monolayer was treated with 9 volumes of a trypsin solution at 37°C for 20 minutes before neutralization with one volume of undiluted FBS and MDMs scraped off the dish. MDMs were seeded in 200 µL of RPMI–10% FBS at 1 × 10^5^ cells per well in 96-well tissue culture plates and incubated for 2 hours for attachment before infection.

### 2.7 MTBC single-cell suspension

**Figure 1:**
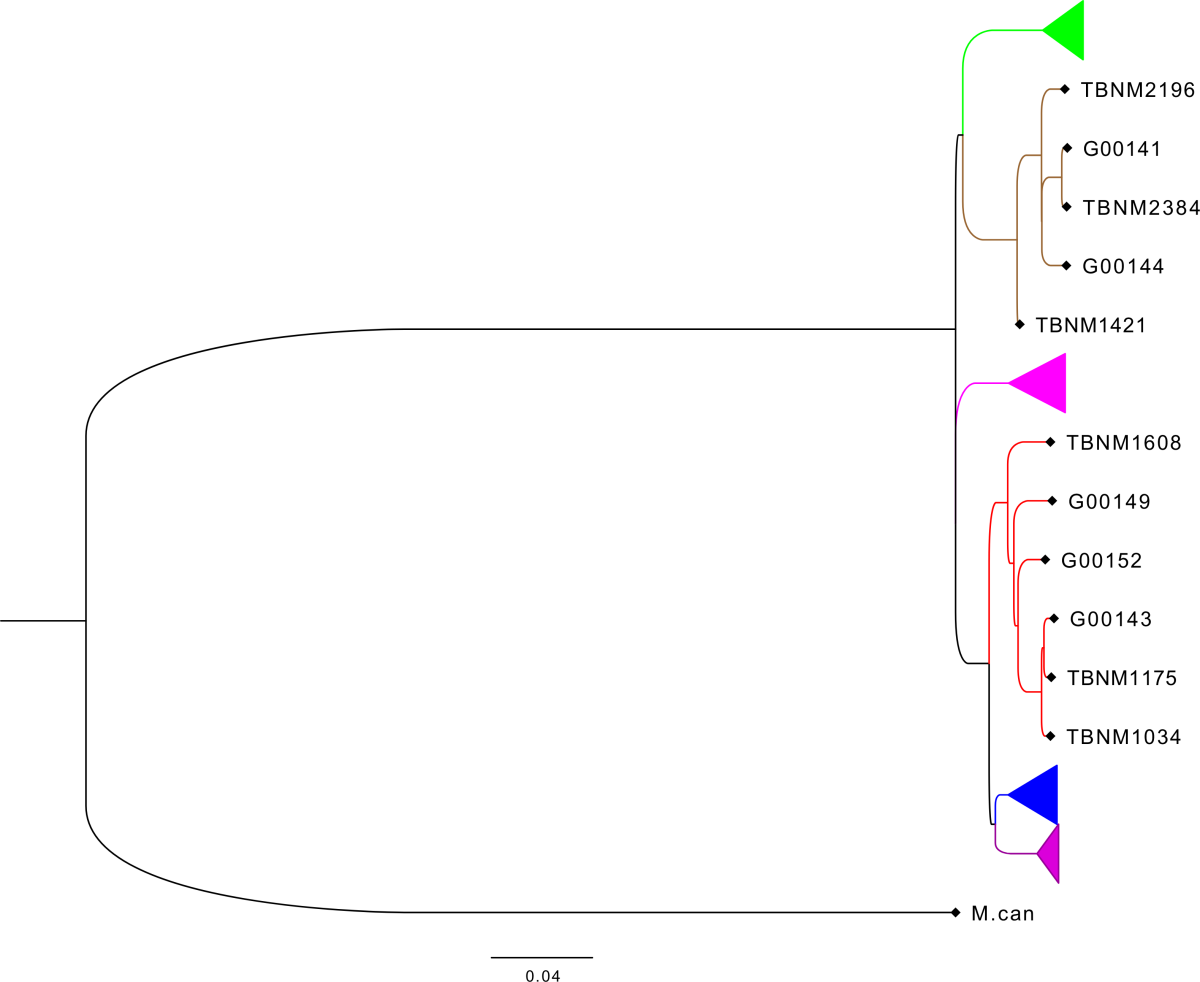
Phylogenetic analysis of mycobacterial isolates used for the study. Phylogenetic tree based on whole-genome sequencing data from L4 (red) and L5 (brown) mycobacterial isolates (TBNM) used for the infection assays as well as other West African sequence data (Genome IDs) and all four other major MTBC lineages (L6 in green, L1 in pink, L2 in blue, and L3 in purple). MTBC, *Mycobacterium tuberculosis* complex.

Previously described drug-susceptible hMTBC isolates (Otchere et al., 2018) including three independent L4 and three independent L5 strains selected from the hMTBC maximum likelihood phylogeny below (**Figure 1**) generated with RAxML as previously described (Otchere et al., 2018) were cultivated in 10% albumin dextrose catalase (ADC)-supplemented 7H9 Middlebrook media containing 0.5% sodium pyruvate (**Table S1**). Single-cell suspensions were prepared using sonication and differential centrifugation as previously reported (Arbués et al., 2020) and stored at −80°C. The colony-forming unit (CFU) content of each mycobacterial isolate preparation was determined following serial dilution and plating on an oleic acid albumin dextrose catalase (OADC)-supplemented 7H11 solid medium.

### 2.8 Macrophage infections and bacterial load assessment

The volume of cryopreserved single-cell suspension required to reach a multiplicity of infection (MOI) of 2 was added onto MDMs (MTBCs L4 and L5 against MDMs from the Akan and Ewe ethnic groups: **Table S2**) and incubated at 37°C in 5% CO_2_ for 2 hours. MDMs from the cured TB cases were infected with MTBC lineages they were previously diagnosed with to establish a sympatric infection assay. MDMs were washed twice with pre-warmed RPMI 1640 to remove non-phagocytosed bacteria. A complete medium was then added for later time points. To assess intracellular bacterial load at indicated time points, MDMs were lysed with 100 µL of 0.1% Triton X-100 in water for 20 minutes, and lysates were serially diluted before plating onto 7H11 agar plates supplemented with 10% OADC and 0.2% glycerol. CFU was assessed after 3–4 weeks of incubation at 37°C.

### 2.9 Fluorescence staining and flow cytometric analyses

**Figure 2:**
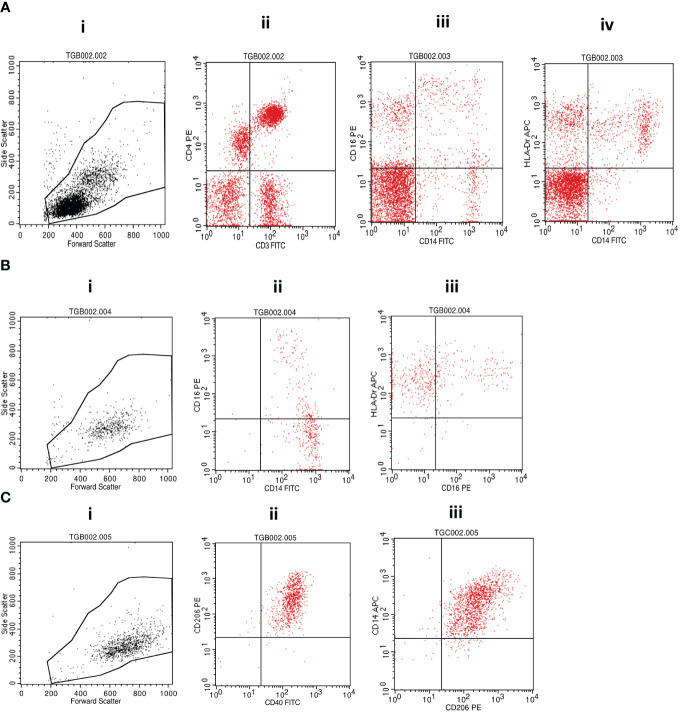
Flow cytometry gating strategy. **(A)** (i) Morphological cell gating on lymphocyte and monocyte based on forward versus side scatter signals was used to determine the representation of (ii) CD3^+^CD4^+^ T cells or (iii) CD14^+^CD16^+^ monocytic cells and (iv) CD14^+^HLA-DR^+^ monocytes within PBMCs. **(B)** Isolated monocytes were analyzed first following morphological gating (i) and then based on their propensity to express (ii) CD14 and/or CD16 and (iii) CD16 and/or HLA-DR. **(C)** Monocyte-derived macrophages were also first analyzed based on their morphology (i) and subsequent fluorescence propensity to express (ii) CD206 and CD40 or (iii) CD14 and CD206. MTBC, *Mycobacterium tuberculosis* complex; PBMCs, peripheral blood mononuclear cells.

Cell surface staining was performed by incubating the investigated cellular fractions with fluorochrome-conjugated antibodies in the dark on ice for 30 minutes before washing with cold phosphate-buffered saline (PBS) and fixing with 10% Cell Fix (250 µL) (BD Biosciences, USA). A cocktail of fluorescently labeled antibodies (BioLegend, San Diego, CA, USA) containing anti-human CD3-FITC (clone HIT3a) and CD4-PE (clone RPA-T4); CD14-FITC (clone: 63D3), CD16-PE (clone: B73.1), and HLA-DR-APC (clone L243); or CD40-FITC (clone 5C3), CD206-PE (clone 15-2) and CD14-APC (clone M5E2) was used for staining. A minimum of 10,000 events were acquired on gated lymphocytes and monocytes on a BD FACSCalibur flow cytometer (BD Biosciences, USA), and data were analyzed using FlowJo software following the gating strategy presented in **Figure 2**.

### 2.10 Data analysis

Figures were constructed and statistical tests including the Mann–Whitney test and two-way analysis of variance (ANOVA) were computed using GraphPad Prism Software, Inc.

## 3 Results

### 3.1 Study participants

A total of 53 participants comprising 20 healthy controls and 33 cured TB patients consented to be enrolled in the study and provided blood for PBMC isolation and phenotyping by flow cytometry (**Table S2**). Out of the total recruited participants, PBMCs from 16 controls and 20 cured TB patients were used for the macrophage infection assay. The median age for the controls was 24.5 years (95% confidence interval: 23–30), and that of the cured TB cases was 32 years (95% confidence interval: 30–43). The controls consisted of eight Akans (female = 4, male = 4) and eight Ewes (female = 3, male = 5), whereas the cured TB cases comprised 11 Akans (female = 1, male = 10) and nine Ewes (female = 4, male = 5) (**Table S3**).

### 3.2 Phenotypic profiling of T cells and isolated PBMCs from controls or cured cases across Ewe and Akan ethnic groups

**Figure 3:**
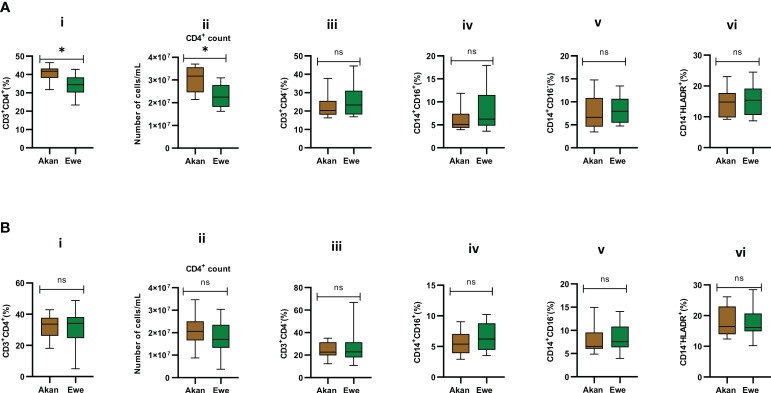
Comparison of the cellular composition of PBMCs from participants. Phenotypic and quantitative characterization of cells in PBMCs of **(A)** healthy controls [Akan, n = 10; Ewe, n = 10] and **(B)** cured TB cases [Akan, n = 18; Ewe, n = 15] of the two ethnic groups. Significant difference was observed only in the proportions (p = 0.02) and counts of CD3^+^CD4^+^ cells (p = 0.02) among the two ethnic groups using Mann–Whitney test. PBMCs, peripheral blood mononuclear cells; TB, tuberculosis. *p-value ≤ 0.05; ns, not significant (statistically), that is, p-value > 0.05.

Median CD4 T-cell frequencies for the two self-reported ethnic groups fell within normal physiological values of 30%–60% for PBMCs (Kleiveland, 2015). However, PBMCs from Akan participants showed significantly higher frequencies and absolute counts of CD4 T cells when compared to Ewe participants with a median frequency of 41.75 and 34.42, respectively (**Figure 3A.i**, p = 0.02). Moreover, the Akan participants showed higher median absolute counts of 3.17 × 10^7^ cells/mL when compared to Ewes with 2.25 × 10^7^ cells/mL (**Figure 3A.ii,** p = 0.02). We observed no significant difference in the proportion of CD3^+^/CD4^−^ T-cell compartment (median: Akan = 20.35, Ewe = 23.33) (**Figure 3A.iii,** p = 0.51). In contrast, the median CD4 T-cell frequencies and absolute counts for the cured TB cases did not differ statistically between self-reported Akan and Ewe ethnic groups (**Figure 3B.i,** p = 0.76; **Figure 3B,ii,** p = 0.23). Also, the CD3^+^/CD4^−^ cell compartment did not show any significant difference between the two ethnic groups (median: Akan = 22.63, Ewe = 22.88) (**Figure 3B.iii,** p = 0.90).

The representation of inflammatory monocytes, CD14^+^/CD16^+^ (**Figures 3A.iv**, **B.iv**), within the isolated PBMC fractions, showed no significant difference between the ethnic groups for both controls (median: Akan = 5.09, Ewe = 6.23, p = 0.39) and cured TB cases (median: Akan = 5.38, Ewe = 6.22, p = 0.32), nor did the classical monocyte compartment, CD14^+^/CD16^−^ (**Figures 3A.v**, **B.v**), for both controls (median: Akan = 6.63, Ewe = 7.96, p = 0.53) and cured TB cases (median: Akan = 6.54, Ewe = 7.58, p = 0.47). Since increasing proportions of CD14^+^/HLA-DR^+^ cells have been associated with increased disease severity (McGill et al., 2022), we sought to also compare their proportions in PBMCs from both controls and cured TB cases for the ethnic groups to eliminate the possible effect of any underlining disease on the study participants (**Figures 3A.vi**, **B.vi**). We observed no statistically significant difference in their frequency for both cohorts, controls (median: Akan = 10.32, Ewe = 12.93, p = 0.11) and cases (median: Akan = 10.66, Ewe = 12.71, p = 0.34).

### 3.3 Phenotypic profiling of sorted monocytes and derived macrophages

**Figure 4:**
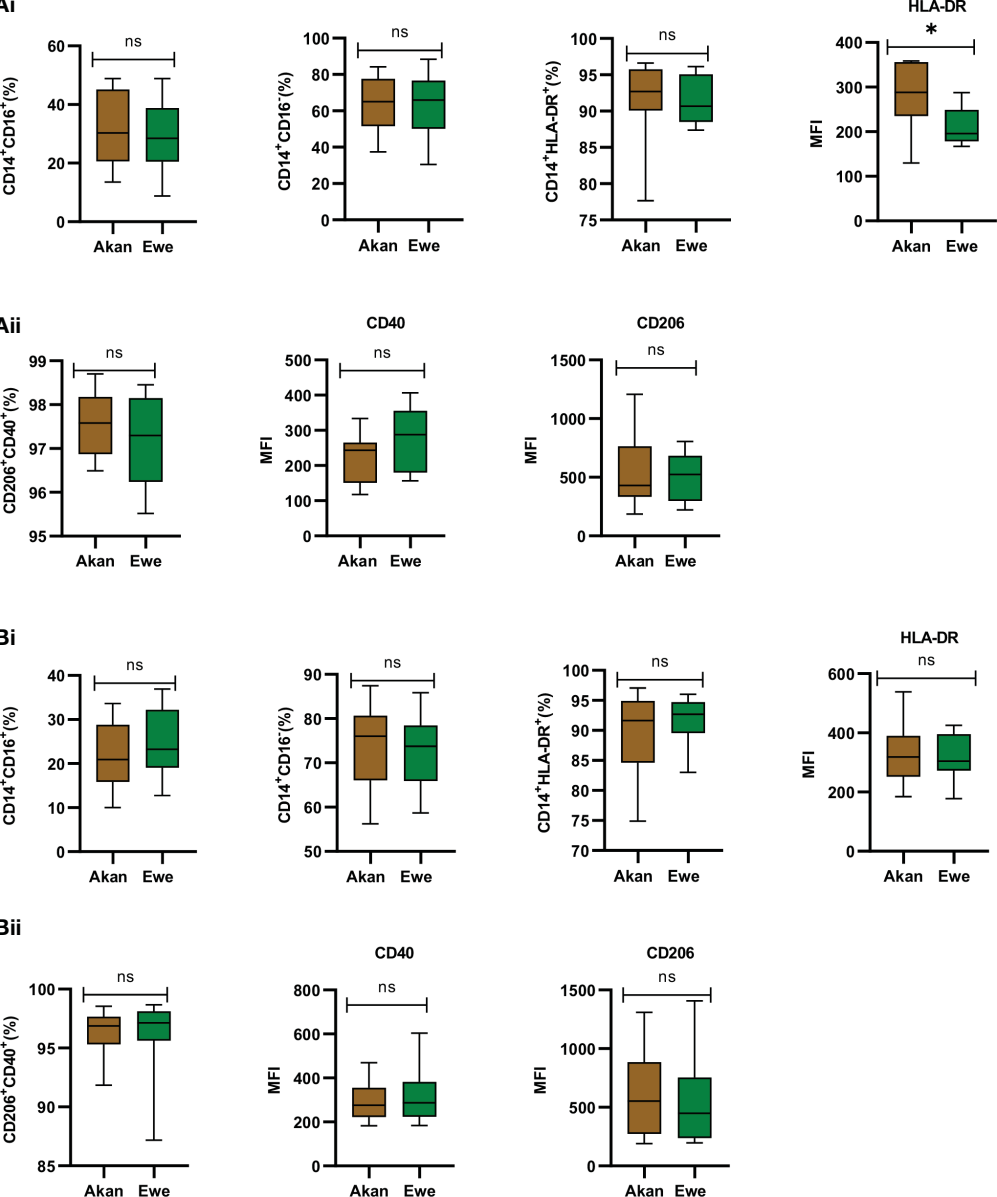
Phenotypic profiling of sorted monocytes and derived macrophages. **(A.i)** Comparison of the proportions of sorted monocytes (CD14^+^CD16^+^, CD14^+^CD16^−^, CD14^+^HLA-DR^+^, and median fluorescent intensity (MFI) of HLA-DR) for controls [Akan, n = 8; Ewe, n = 8]. **(A.ii)** Comparison of derived macrophages (CD206^+^CD40^+^ and MFI of CD40 and CD206) for controls. **(B.i)** Comparison of the proportions of sorted monocytes (CD14^+^CD16^+^, CD14^+^CD16^−^, CD14^+^HLA-DR^+^, and MFI of HLA-DR) for cured TB cases [Akan, n = 11; Ewe, n = 9] across ethnicity. **(B.ii)** Comparison of the proportions of macrophages (CD206^+^CD40^+^ and MFI of CD40 and CD206) among the ethnic groups for cured TB cases. A significant difference was seen for MFI of HLA-DR of the controls (p = 0.03) using Mann–Whitney test. TB, tuberculosis. *p-value ≤ 0.05; ns, not significant (statistically), that is, p-value > 0.05.

Sorted monocytes were analyzed by flow cytometry to assess their purity and composition before differentiation into macrophages (**Figures 4A.i, B.i**). CD14^+^ fractions’ purity was systematically above 90% and composed of equal representation across study groups by classical CD14^+^/CD16^−^ (controls: Akan = 65.02%, Ewe = 65.96%, p = 0.91; cases: Akan = 76.05%, Ewe = 73.77%, p = 0.85) and CD14^+^/CD16^+^ inflammatory monocytes (controls: Akan = 30.28, Ewe = 28.46, p = 0.85; cases: Akan = 20.92, Ewe = 23.27, p = 0.42). The proportion of HLA-DR-expressing monocytes was also found constant (controls: Akan = 92.69, Ewe = 90.68, p = 0.45; cases: Akan = 91.64, Ewe = 92.67, p = 0.53) across the ethnic groups for both controls and cured TB cases.

Consequently, the phenotype of MDMs expressing CD40, which are responsible for the production of pro-inflammatory cytokines and nitric oxide upon stimulation (Danese et al., 2004), and CD206, which function in phagocytosis and immune homeostasis (Azad et al., 2014), were also found homogenous across ethnic groups for controls (median: Akan = 97.58; Ewe = 97.30, p = 0.66) (**Figure 4A.ii**) and cases (median: Akan 96.88; Ewe = 97.18, p = 0.32) (**Figure 4B.ii**).

As reflected by an increased mean fluorescence intensity (MFI) of the HLA-DR staining, the expression of MHC-II molecules by monocytes from Akan control blood donors (median = 288.3) was significantly higher than that of Ewes (median = 195.9) (p = 0.03) (**Figure 4A.i**). However, this observation was no longer found among cured TB cases (median: Akan = 318.5, Ewe = 304.9, p = 0.76) (**Figure 4B.i**). Analysis of CD40 and CD206 MFI revealed no significant difference across ethnicity for both controls (CD40: Akan = 243.6, Ewe = 287.8, p = 0.18; CD206: Akan = 429.4, Ewe = 523.3, p = 0.73) and cases (CD40: Akan = 276.3, Ewe = 287.7, p = 0.91; CD206: Akan = 552.3, Ewe = 450.0, p = 0.43).

### 3.4 Growth assessment of L4 and L5 in macrophages of the same ethnic group

**Figure 5:**
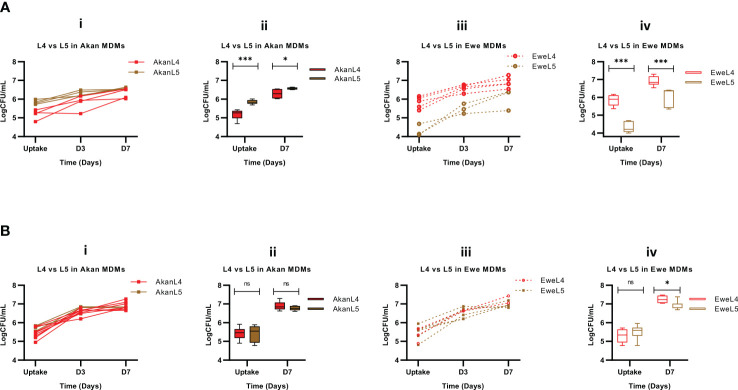
Growth assessment of L4 and L5 in control macrophages **(A)** [Akan, n = 8; Ewe, n = 8] and cured TB cases **(B)** [Akan, n = 11; Ewe, n = 9]. (i) Comparison of growth of L4 and L5 at uptake, day 3 (D3), and day 7 (D7) in Akan macrophages. (ii) Comparison of the growth rate of L4 and L5 in Akan macrophages between uptake and D7. (iii) Comparison of the growth of L4 and L5 in Ewe macrophages (iv) using multiple Mann–Whitney test. TB, tuberculosis. *p-value ≤ 0.05; ***p-value ≤ 0.001; ns, not significant (statistically), that is, p-value > 0.05.

The first analysis was carried out to assess the growth of either L4 or L5 MTBC strains in macrophages derived from monocytes from the same ethnic group. Compared to L4 strains, L5 strains resulted in higher bacillary loads 7 days post-infection in Akan macrophages of control participants (p < 0.0001, **Figure 5A.i**). This difference seems to reflect an increased capacity of Akan macrophages to phagocytose L5 strains, as the intracellular bacterial load was already statistically superior at uptake (p < 0.001) and was still higher at D7 (p = 0.04) (**Figure 5A.ii**). However, the replication of L4 strains compared to L5 in Akan macrophages was 1.2 versus 1.1, respectively (p = 0.04).

In contrast, Ewe macrophages from healthy controls (**Figure 3A.iii**) showed a higher growth for L4 (p < 0.0001) linked to a statistically significant difference at uptake (p < 0.001) (**Figure 5A.iv**). Nevertheless, the replication of L5 strains in Ewe macrophages was higher than that of L4 strains (fold change: L5 = 1.4, L4 = 1.2, p = 0.03).

Further analysis of the growth of L4 and L5 in Akan macrophages of the cured TB cases showed no significant difference (p = 0.49) (**Figure 5B.i**). Moreover, there was no difference in the log_10_CFU/mL at uptake (p = 0.71) and D7 (p = 0.27) (**Figure 5B.ii**), and the replication of L4 and L5 strains was 1.3 and 1.2, respectively (p = 0.50). We observed similar patterns with the growth comparison of the two lineages in Ewe macrophages of the cured TB cases where there was no significant difference in bacillary load (**Figure 5B.iii**) (p = 0.99). There was no difference in the uptake of L4 and L5 (p = 0.48), but there was a significant difference in the growth of L4 at D7 (p = 0.02) (**Figure 5B.iv**) with a fold change of 1.4 for L4 and 1.3 for L5 (p = 0.08).

### 3.5 Growth assessment in macrophages from Akans and Ewes

**Figure 6:**
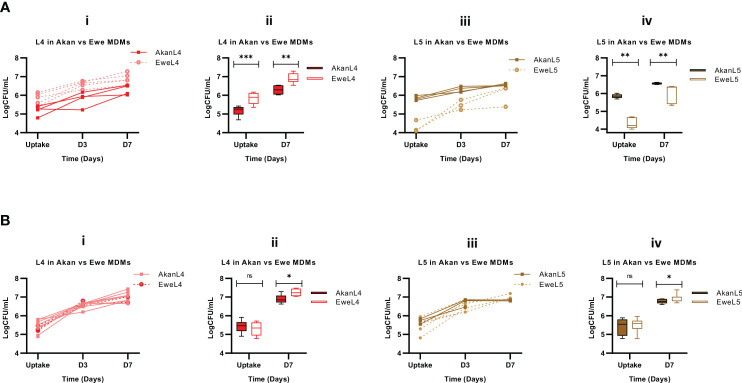
Comparison of Akan and Ewe macrophages from control **(A)** [Akan, n = 8; Ewe, n = 8] and cured TB cases **(B)** [Akan, n = 11; Ewe, n = 9] in support of MTBC growth. (i) Comparison of the growth of L4 at uptake, D3, and D7 in Akan and Ewe macrophages. (ii) Comparison of the growth rate of L4 in Akan and Ewe macrophages between uptake and D7. (iii) Comparison of the growth of L5 in Akan and Ewe macrophages. (iv) Growth rate comparison of L5 in Akan and Ewe macrophage between uptake and D7 using multiple Mann–Whitney test. TB, tuberculosis; MTBC, *Mycobacterium tuberculosis* complex. *p-value ≤ 0.05; **p-value ≤ 0.01; ***p-value ≤ 0.001; ns, not significant (statistically), that is, p-value > 0.05.

We then sought to stratify our analysis by comparing the influence of macrophage donors’ ethnicity on the uptake and subsequent growth of L4 and L5 strains. This analysis revealed that L4 strains achieved higher bacterial load in macrophages derived from Ewe control donors in comparison to Akans (p < 0.0001, **Figure 6A.i**), reflecting a significantly higher bacterial uptake 2 hours post-infection (p < 0.0001) and higher bacterial growth 7 days (p < 0.001) post-infection (**Figure 6A.ii**). However, the replication of L4 in Akan versus Ewe macrophages was comparable (fold change: 1.2, p = 0.85). In contrast, L5 strains in macrophages derived from Ewes displayed significantly lower uptake (p < 0.001, **Figure 6A.iv**) that also translated into reduced intracellular bacterial load 7 days post-infection (p < 0.0001). Hence, Akan macrophages obtained from healthy controls supported the uptake of L5 and consequently higher bacterial load at day 7 than Ewe macrophages. On the contrary, the bacterial replication of L5 in Ewe macrophages was higher than that of Akans (fold change: L5 in Ewe MDMs = 1.4, L5 in Akan MDMs = 1.1, p = 0.004).

Further analysis among the cured TB cases showed that L4 strains displayed similar uptake in both macrophages after 2 hours of infection (p = 0.48); however, there was a higher bacterial load in Ewe macrophages compared to that of the Akans at day 7 (p = 0.02) (**Figure 6B.ii**) with a fold change of 1.3 in Akan macrophages and 1.4 in Ewe macrophages (p = 0.03). Similarly, we observed a significantly higher bacterial load of L5 strains in Ewes macrophages at day 7 post-infection (p = 0.02) and no significant difference at uptake (p = 0.67) (**Figure 6B.iv**). The fold change of L5 in Ewe macrophages was 1.3, whereas that of Akans’ was 1.2 (p = 0.55). Ewe macrophages from the cured TB cases supported the replication of L4 strains better than Akan macrophages.

## 4 Discussion

We aimed to investigate the early phase of the pathogenesis of representative MTBC strains endemic in Ghana that could sustain our previously observed epidemiological findings. Akans, the largest ethnic group in Ghana, are mostly affected by MTBC-L4 infections and to a lesser extent by L5 and L6 MTBC strains. In return, individuals that self-reported to belong to the Ewe ethnic group were described to be ~3 times more likely to be affected by MTBC-L5 infections (Asante-Poku et al., 2015; Asante-Poku et al., 2016). We hypothesized that such epidemiological linkage may translate into the differential capacity of macrophages from these two ethnic groups to endure intracellular infections *in vitro*. To test our hypothesis, we performed *ex vivo* infections of monocyte-derived macrophages from the blood of donors belonging to the above-mentioned ethnic groups that were found to be epidemiologically linked with specific MTBC lineages isolated in Ghana.

Considering data obtained from the blood of healthy controls, Ewe macrophages displayed a superior propensity to phagocytose L4 bacilli when compared to Akan MDMs. However, the replication of L4 was higher in Akan MDMs, which is consistent with the previous epidemiological observation that L4 is associated with the Akan ethnic group. In contrast, we observed increased uptake of L5 by Akan MDMs. Despite the lower uptake of L5 by Ewe MDMs, the bacterial load measured 1 week post-infection resulted in a substantially higher replication rate of L5 in macrophages derived from Ewe compared to Akan blood donors. This observation also corroborates findings from epidemiological studies where L5 infections were associated with the Ewe ethnic group. However, the uptake observed was the reverse of the mycobacterial growth in the macrophages. This shows a possible co-evolution of phagocytosis of MTBC by macrophages of different ethnicity. Several pathogens have evolved subtle strategies to evade phagocytosis or induce reduced phagocytosis as a virulence mechanism (Uribe-Querol and Rosales, 2017). MTBC L4 might have co-evolved with Akan macrophages where they have both mechanisms to reduce phagocytosis and increase intracellular growth, whereas L5 has also developed mechanisms to reduce phagocytosis and increase its growth within Ewe macrophages.

The analysis of data generated with blood from cured TB patients did not reveal the same differential uptake between the two ethnic groups. However, MDMs from Ewes displayed a mild but significant tendency to allow higher multiplication rates for both L4 and L5 strains. These results suggest that host ethnicity, MTBC genetic diversity, and trained innate immunity mediated by recent infection would all be acting in concert to modulate the outcome of macrophage infections.

Indeed, recent studies have reported the ability of trained innate immunity to impact MTBC infection outcomes (Zhou et al., 2021). Some individuals who are highly exposed to TB do not develop the disease nor latent infection due to early clearance attributed to *Mycobacterium bovis* BCG exposure. BCG vaccination boosts innate immune responses through epigenetic remodeling. Similarly, previous TB infections also promote innate immunity training (Koeken et al., 2019; Ferluga et al., 2020). Taken together, we propose that trained innate immunity due to recent TB eliminates the effect of self-reported ethnicity observed in healthy controls and induces similar outcomes in the phagocytosis capacity of MDMs independently of self-reported ethnicity. As a limitation of this study, information on the BCG vaccination of the participants was not obtained for further analysis of its impact on trained immunity. However, the role of pathogen virulence and possibly host genetic background could reduce the effect of trained immunity. This was observed in the higher growth of L4 even among the cured TB cases and the general higher growth of MTBC in Ewe macrophages. Thus, diversity in macrophage infection with MTBC could be multifactorial.

The higher replication rate of L5 in Ewe macrophages but lower phagocytosis of L5 in Ewe macrophages supports our hypothesis of a possible host–pathogen co-evolution. We propose that L5 strains have co-evolved with the Ewe ethnic group, resulting in the sympatric association observed nowadays, which translates experimentally into an increased intracellular replication propensity. Nonetheless, a lower bacillary load can be associated with the fact that recovery may be suboptimal due to differentially cultivable mycobacteria associated with non-replicative dormant states. MTBC is able to adapt to environmental conditions such as hypoxia and reduced nutrients presented in macrophages and progress to a non-replicative dormant state (Gengenbacher and Kaufmann, 2012). L5 and L6 strains have been associated with a higher tendency to progress into a dormant state when compared to L4 (Ofori-Anyinam et al., 2017).

Macrophages have been reported to respond to hypoxia and other environmental conditions differentially, which is very relevant to the understanding of disease mechanisms. Low oxygen tension has been associated with changes in macrophage morphology, phagocytosis, release of cytokines, and metabolic activities (Lewis et al., 1999). Macrophages from the different ethnic groups might be responding differently to comparable environmental conditions, hence resulting in the diversity in phagocytic abilities of the macrophages obtained from donors of the two ethnic groups.

Phenotypic studies on *in vitro* growth assessment revealed slower growth of L5 strains compared to L4 isolates (Gehre et al., 2013; Ates et al., 2018; Osei-Wusu et al., 2021). The slow growth of L5 has been attributed to its attenuated virulence. Due to the unique phenotypic characteristics of L5, it was expected to be outcompeted by more virulent lineages such as L4. However, it has remained a relevant causative agent for TB in West Africa over the years (Yeboah-Manu et al., 2016). The *ex vivo* infection data presented here suggest that L5 strains may replicate as well as L4 isolates once inside their natural host cell and so particularly when cells originated from the self-reported Ewe ethnic group. This observation could explain the unexplained prevalence of L5 infections in West Africa over the years. Host genetics is beginning to provide answers in TB host–pathogen interplays. For instance, it was reported that 5-lipoxygenase (*ALOX5*) variants are associated with ethnicity and susceptibility to TB in Ghana (Herb et al., 2008). Another recent study in Ghana on host susceptibility to TB has suggested two potentially protective genes, SLC11A1 and SORBS2 (Asante-Poku et al., 2022). Further investigations looking into the distribution of genetic variants in Ewes compared to other Ghanaian ethnic groups are needed to further our mechanistic understanding of the macrophage phenotypes presented in this study.

## 5 Limitation

The MTBC lineage diversity observed could have been a result of the individual strain effect, which is very prominent among MTBC. Different strains of MTBC may differ in virulence and immunogenicity. However, we have carefully selected strains representative of the two most prevalent L4 sub-lineages (Cameroon and Ghana sub-lineages) and the most prevalent L5 sub-lineages to attempt to circumvent this limitation. However, gender distribution was a challenge since we could recruit equal numbers for the cured TB cases.

Although our study may not provide a very conclusive explanation of the epidemiological association of specific MTBC lineages with ethnicity in Ghana, it provides insight into this relationship and serves as the platform for future studies.

## 6 Conclusion

We observed that Ewe MDMs demonstrated a higher propensity to phagocytose L4 strains compared to Akan MDMs; nevertheless, the replication rate of L4 was higher in Akan MDMs. In contrast, we observed an increased uptake of L5 by Akan MDMs, despite the substantially higher replication rate of L5 in Ewe MDMs compared to that of Akans. Interestingly, the impact of self-reported ethnicity disappeared when using cells derived from the blood of cured TB cases. In return, Ewe MDMs appeared more susceptible to both L4 and L5 strains. These results suggest that host ethnicity and, by extension, host genetic diversity together with MTBC genetic diversity and previous exposure to MTBC are all acting together to modulate macrophage invasion and intracellular replication. Further studies are required to explain the mechanism underlying the observation while considering host genetic, social, and nutritional factors. Also, with the observed diversity in CD4 T cells of the controls, we would like to study their activation, maturation, and exhaustion in a detailed study in the future. Moreover, it is important to study the interaction of surface markers of the macrophages with the different lineages of MTBC to further understand the mechanism of the higher phagocytic properties of the Akan macrophages for L5 and Ewe macrophages for L4. Studies on the cytokine profiles of the infection assay are recommended.

## Data availability statement

The original contributions presented in the study are included in the article/**Supplementary Material**, further inquiries can be directed to the corresponding author/s.

## Ethics statement

The studies involving human participants were reviewed and approved by Institutional Review Boards (IRB) of Korle-Bu Teaching Hospital (KBTH) and Noguchi Memorial Institute for Medical Research (NMIMR), University of Ghana (Federal wide assurance number: FWA00001824). Written informed consent to participate in this study was provided by the participants’ legal guardian/next of kin.

## Author contributions

All authors contributed to the article and approved the submitted version.
