# Supplementary material for: Macrophage susceptibility to infection by Ghanaian Mycobacterium tuberculosis complex lineages 4 and 5 varies with self-reported ethnicity
Source: Front Cell Infect Microbiol. 2023 Aug 14;13:1163993. doi: 10.3389/fcimb.2023.1163993 (PMC10461633; doi:10.3389/fcimb.2023.1163993)
Supplement: Supplementary file 1 [file Table_1.docx]

**Supplementary Tables**

**Table S1: Mycobacterial isolates used for single cell suspension**

| **Isolate ID(TB/NM)** | **Lineage** | **Sub-lineage** | **G-Number** | **Accession**  **number** |
| --- | --- | --- | --- | --- |
| 1034 | L4 | Cameroon | G33068 | SRR11444325 |
| 1175 | L4 | Cameroon | G08638 | ERR502518 |
| 1608 | L4 | Ghana | G32790 | SRR11444335 |
| 1421 | L5 | L5.1 | G08700 | ERR751300 |
| 2196 | L5 | L5.3 | G08711 | ERR751311 |
| 2384 | L5 | L5.4 | G08490 | ERR1082139 |

**Table S2: Sources of monocyte derived macrophages with their corresponding infecting mycobacterial isolates**

| **Healthy controls** | | | | **Cured TB patients** | | | |
| --- | --- | --- | --- | --- | --- | --- | --- |
| **Participant ID** | **Ethnicity** | **Isolate ID** | **Lineage** | **Participant ID** | **Ethnicity** | **Isolate ID** | **Lineage** |
| TGC 008 | Akan | 1034 | L4 | TGB 006 | Akan | 1034 | L4 |
| TGC 009 | Akan | 1034 | L4 | TGB 012 | Akan | 1034 | L4 |
| TGC 013 | Akan | 1034 | L4 | TGB 016 | Akan | 1608 | L4 |
| TGC 012 | Akan | 1175 | L4 | TGB 020 | Akan | 1034 | L4 |
| TGC 015 | Akan | 1421 | L5 | TGB 022 | Akan | 1034 | L4 |
| TGC 023 | Akan | 1421 | L5 | TGB 023 | Akan | 1175 | L4 |
| TGC 022 | Akan | 1421 | L5 | TGB 026 | Akan | 1175 | L4 |
| TGC 021 | Akan | 1421 | L5 | TGB 031 | Akan | 1608 | L4 |
| TGC 011 | Ewe | 1175 | L4 | TGB 017 | Akan | 2196 | L5 |
| TGC 019 | Ewe | 1034 | L4 | TGB 030 | Akan | 1421 | L5 |
| TGC 018 | Ewe | 1034 | L4 | TGB 044 | Akan | 2384 | L5 |
| TGC 016 | Ewe | 1034 | L4 | TGB 014 | Ewe | 1034 | L4 |
| TGC 017 | Ewe | 1034 | L4 | TGB 028 | Ewe | 1034 | L4 |
| TGC 002 | Ewe | 2196 | L5 | TGB 037 | Ewe | 1175 | L4 |
| TGC 001 | Ewe | 2196 | L5 | TGB 008 | Ewe | 2196 | L5 |
| TGC 010 | Ewe | 2196 | L5 | TGB 019 | Ewe | 1421 | L5 |
|  |  |  |  | TGB 024 | Ewe | 1421 | L5 |
|  |  |  |  | TGB 040 | Ewe | 2384 | L5 |
|  |  |  |  | TGB 045 | Ewe | 2384 | L5 |
|  |  |  |  | TGB 047 | Ewe | 2384 | L5 |

**Table S3: Demography of study participants**

|  |  | **Flow cytometry** | | | | **Macrophage infection assay** | | | |
| --- | --- | --- | --- | --- | --- | --- | --- | --- | --- |
|  |  | **Controls**, n=20 | | **Cured cases**, n=33 | | **Controls**, n=16 | | **Cured cases**, n=20 | |
|  |  | Akan n=10 | Ewe n=10 | Akan  n=18 | Ewe n=15 | Akan n=8 | Ewe n=8 | Akan n=11 | Ewe n=9 |
| **Gender** | Male | 5 | 5 | 13 | 11 | 4 | 5 | 10 | 5 |
|  | Female | 5 | 5 | 5 | 4 | 4 | 3 | 1 | 4 |
| **Age** | < 15 | - | - | - | - | - | - | - | - |
|  | 15-30 | 9 | 9 | 5 | 3 | 7 | 7 | 4 | 1 |
|  | 31-45 | - | 1 | 7 | 8 | 1 | - | 4 | 6 |
|  | 46-60 | 1 | - | 4 | 3 | - | 1 | 2 | 2 |
|  | > 60 | - | - | 2 | 1 | - | - | 1 | - |
